# A Multicenter Pivotal Study on the Artificial Intelligence System for Neoplastic Lesions Detection in Upper Gastrointestinal Endoscopy

**DOI:** 10.1111/den.70015

**Published:** 2025-09-12

**Authors:** Seiichiro Abe, Yoshiyasu Kitagawa, Waku Hatta, Takao Maekita, Motohiko Kato, Akihito Nagahara, Hiroyuki Osawa, Osamu Dohi, Hirotaka Nakashima, Kazuhiro Furukawa, Shiro Oka, Tomoko Yokoyama, Toru Ito, Ichiro Oda

**Affiliations:** ^1^ Endoscopy Division National Cancer Center Hospital Tokyo Japan; ^2^ Endoscopy Division Chiba Cancer Center Chiba Japan; ^3^ Division of Gastroenterology Tohoku University Graduate School of Medicine Sendai Japan; ^4^ Second Department of Internal Medicine Wakayama Medical University Wakayama Japan; ^5^ Center for Diagnostic and Therapeutic Endoscopy Keio University School of Medicine Tokyo Japan; ^6^ Department of Gastroenterology Juntendo University Faculty of Medicine Tokyo Japan; ^7^ Division of Gastroenterology, Department of Medicine Jichi Medical University Shimotsuke Japan; ^8^ Molecular Gastroenterology and Hepatology Graduate School of Medical Science, Kyoto Prefectural University of Medicine Kyoto Japan; ^9^ Department of Gastroenterology Foundation for Detection of Early Gastric Carcinoma Tokyo Japan; ^10^ Cancer Screening Center, Cancer Institute Hospital of Japanese Foundation for Cancer Research Tokyo Japan; ^11^ Department of Gastroenterology and Hepatology Nagoya University Graduate School of Medicine Nagoya Japan; ^12^ Department of Gastroenterology Hiroshima University Hospital Hiroshima Japan; ^13^ FUJIFILM Nishiazabu Endoscopy Clinic Tokyo Japan; ^14^ Department of Gastroenterological Endoscopy Kanazawa Medical University Hospital Kahoku Japan; ^15^ Department of Internal Medicine Kawasaki Rinko General Hospital Kawasaki Japan

**Keywords:** artificial intelligence, detection, endoscopy, esophageal cancer, gastric cancer

## Abstract

**Objectives:**

This pivotal study aimed to evaluate the performance of the CAD‐EYE prototype in identifying esophageal squamous cell carcinoma (ESCC) and gastric neoplasm (GN) for regulatory approval of the Pharmaceuticals and Medical Devices Agency in Japan.

**Methods:**

This retrospective study utilized image datasets comprising 15 consecutive video frames captured using non‐magnifying white‐light imaging (WLI), blue laser/light imaging (BLI), and linked color imaging (LCI). The sensitivity and specificity of the CAD‐EYE prototype for successful detection were calculated using the gold standard, which consists of image datasets of neoplastic lesions annotated by experienced endoscopists.

**Results:**

A total of 620, 679, and 682 ESCC datasets were analyzed in the WLI, BLI, and LCI groups, respectively. The sensitivity and specificity of ESCC detection were 85.9% [81.1%–90.6%] and 93.3% [90.8%–95.7%] in the WLI group, 97.6% [95.6%–99.7%] and 92.9% [90.6%–95.3%] in the BLI group, and 96.6% [94.2%–99.1%] and 93.2% [91.0%–95.5%] in the LCI group. The sensitivities for pT1a ESCC were 85.3%, 97.3%, and 97.2% in the WLI, BLI, and LCI groups, respectively. For GN, 841 WLI and 882 LCI datasets were analyzed. The sensitivity, specificity, and specificity in the detection flag of GN detection were 95.5% [92.8%–98.1%], 86.1%, and 85.4% [82.6%–88.2%] in the WLI group, and 93.9% [91.1%–96.7%], 94.4%, and 93.9% [92.0%–95.8%] in the LCI group, respectively. The sensitivities for pT1a early gastric cancer were 93.8% and 92.4% in the WLI and LCI groups, respectively.

**Conclusions:**

The CAD‐EYE prototype demonstrated high sensitivity in detecting ESCC and GN, highlighting its potential as a promising tool for clinical applications.

## 1 Introduction

Gastric and esophageal cancers are the fourth and seventh most common cancers worldwide, accounting for 8.2% and 5.3% of all cancer diagnoses, respectively [1]. Early detection and treatment are crucial to improve the prognosis of these cancers [2]. Endoscopic resection is an established minimally invasive treatment for early gastric cancer (EGC) and superficial esophageal squamous cell carcinoma (ESCC) with a low risk of lymph node metastasis. This approach allows for organ preservation and provides favorable long‐term outcomes [3, 4]. In Asian countries, esophagogastroduodenoscopy is the primary modality used for gastric cancer screening [5, 6]. Additionally, a community‐based allocation trial in an endemic area of China demonstrated a 33% reduction in cumulative ESCC‐related mortality in a 10‐year follow‐up in the intervention group compared to the unscreened group [7].

White‐light imaging (WLI) is the most commonly used modality for the endoscopic observation of the stomach [8]. However, achieving satisfactory endoscopic detection of gastric neoplasms (GN) such as EGC and gastric adenoma requires a high level of expertise. Several studies have reported gastric cancer miss rates ranging from 4.6% to 25.8% [9, 10, 11, 12, 13, 14]. Image‐enhanced endoscopy (IEE) techniques, such as narrow‐band imaging (NBI) and blue laser/light imaging (BLI), have shown utility in detecting EGC [15]. BLI is a widely used modality, similar to NBI, for detecting lesions in the esophagus [16, 17]. Linked color imaging (LCI) is another advanced imaging technique that enhances the visual differentiation of mucosal color by simultaneously expanding and contracting the color spectrum between red and white. A recent randomized controlled trial showed that LCI is more effective than high‐definition WLI in detecting neoplastic lesions in the pharynx, esophagus, and stomach [18].

Artificial intelligence (AI) has recently made remarkable progress, and computer‐aided detection (CAD) system for gastrointestinal endoscopy is rapidly evolving. The development of AI software that provides real‐time information displayed directly on an endoscopic screen is expected to aid in the detection of neoplastic lesions. Furthermore, the integration of AI with virtual chromoendoscopy such as BLI or LCI is expected to improve the accuracy of detecting early‐stage cancer lesions. Based on this background, Fujifilm recently developed a CAD system (CAD EYE, Fujifilm, Tokyo, Japan) for the detection of gastrointestinal neoplasms using WLI and IEE. This pivotal study aims to evaluate the performance of the CAD‐EYE prototype in detecting ESCC and GN for regulatory approval of the Pharmaceuticals and Medical Devices Agency in Japan.

## 2 Methods

### 2.1 CAD EYE

**FIGURE 1:**
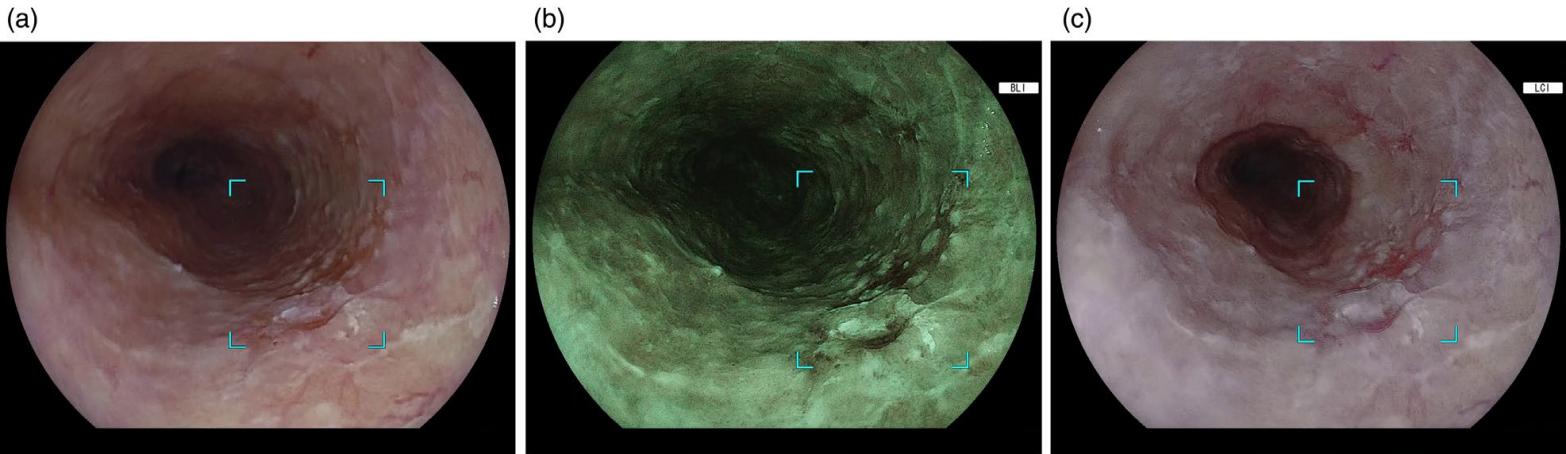
Endoscopic images of esophageal squamous cell carcinoma. (a) Esophageal squamous cell carcinoma identified by CAD EYE with a rectangular frame on WLI. (b) Esophageal squamous cell carcinoma identified by CAD EYE with a rectangular frame on BLI. (c) Esophageal squamous cell carcinoma identified by CAD EYE with a rectangular frame on LCI.

**FIGURE 2:**
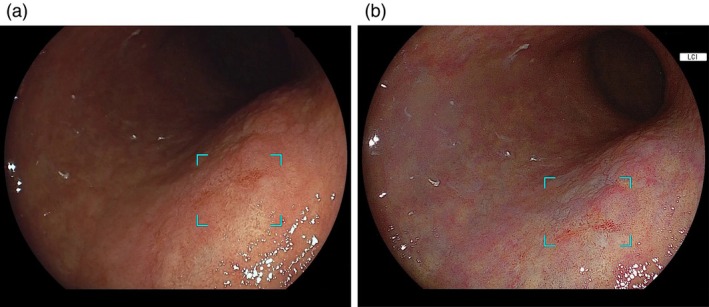
Endoscopic images of gastric neoplasm. (a) Early gastric cancer identified by CAD EYE with a rectangular frame on WLI. (b) Early gastric cancer identified by CAD EYE with a rectangular frame on LCI.

The CAD EYE (EW10‐EG01 prototype, Fujifilm) is a CAD system that employs deep learning technology to analyze video images of neoplastic and non‐neoplastic lesions in the esophagus and stomach. The system was trained using a dataset of more than 250 000 non‐magnifying still images owned by Fujifilm Corporation (Tokyo, Japan), collected under the EG01 (Fujifilm cooperation) protocol for developing CAD systems from 14 Japanese institutions. The CAD EYE receives non‐magnifying endoscopic image data from an image processor and identifies neoplastic lesion candidates by generating an audible alert and visually marking the lesion location with a rectangular frame displayed on the monitor. If multiple lesions are present, each is outlined with a separate rectangle. This CAD EYE prototype is compatible with WLI, BLI, and LCI for detecting ESCC and with WLI and LCI for detecting GN (Figures 1 and 2). BLI is a virtual chromoendoscopy technology integrated into the endoscopic processor developed by Fujifilm Corporation. Blue laser light is used in the laser light source system, and a blue light‐emitting diode is integrated into the light‐emitting diode light source system. Both light sources use a 410‐nm narrow‐band wavelength to provide high‐contrast visualization of microvessels and surface structures of the lesions. LCI is also installed in both the laser and light‐emitting diode systems. In addition to the narrow‐band wavelength of 410 nm, LCI incorporates image processing that improves color separation between red and white, enhancing the detection of subtle mucosal color differences.

### 2.2 Study Subjects

This multicenter, retrospective, video‐based observational study was conducted at 14 referral institutions in Japan. Patients with available endoscopic imaging datasets, with or without ESCC or GN, were included. ESCC was pathologically confirmed according to the Japanese classification of esophageal cancer, while GN was defined as pathologically proven gastric adenoma or gastric cancer, as per the Japanese classification of gastric cancer [19, 20]. In these classifications, high‐grade dysplasia based on World Health Organization classification is considered intramucosal cancer [21]. Endoscopies were performed using non‐magnifying WLI, BLI, or LCI at their discretion. Collected data included the macroscopic type and lesion size based on endoscopic diagnosis. Esophagogastroscopy was performed using high‐definition esophagogastroscopes (EG‐L600ZW7, EG‐L600WR7, EG‐L580RD7, EG‐L580NW7, EG‐760Z, EG‐760R, EG‐740N, EG‐6600Z, EG‐6600R, and EG‐6400N) combined with image processors (VP‐7000, LL‐7000, BL‐7000, and EP‐6000). All procedures were prospectively video‐recorded at the participating institutions. Neoplastic lesions were subsequently resected, either endoscopically or surgically, and confirmed histologically. This study was approved by the institutional review boards of all participating facilities, and written informed consent for this study was obtained from all patients.

### 2.3 The Image Dataset

**FIGURE 3:**
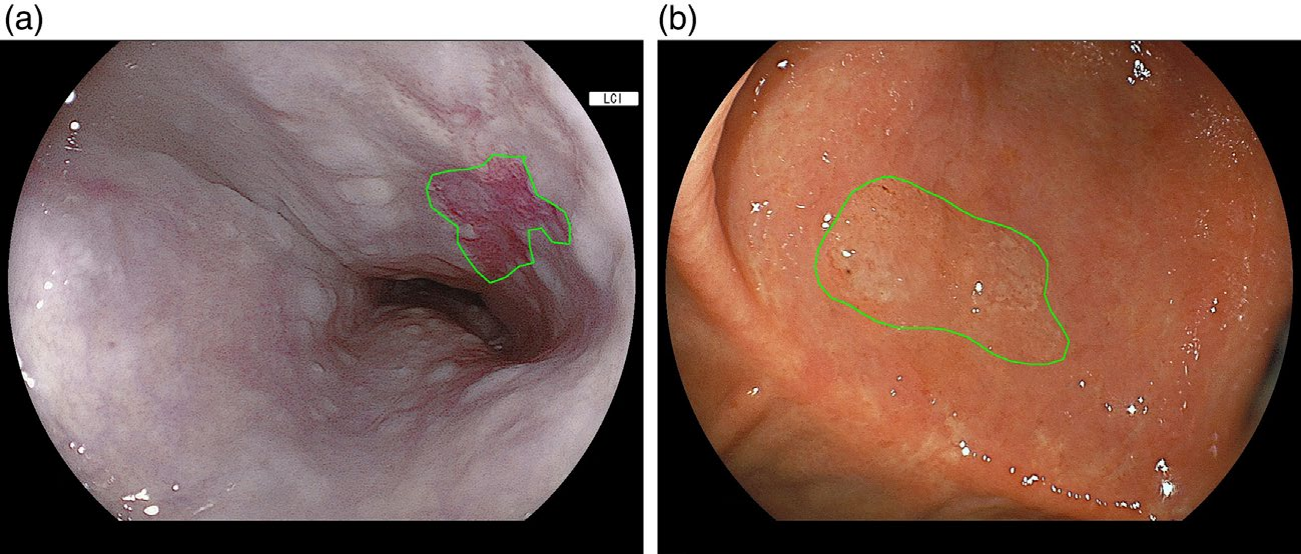
Gold standard of neoplastic lesion. (a) Esophageal squamous cell carcinoma with its boundary annotated as the gold standard. (b) Early gastric cancer with its boundary annotated as the gold standard.

The still images and videos used in this study were anonymized and sent to a contract research organization (CRO; Mediscience Planning Inc., Tokyo, Japan). The CRO created a video clip consisting of 15 consecutive frames (captured at 60 fps) with or without neoplastic lesions from the recorded video, using still images captured by the endoscopist as a reference. The video clip was evaluated offline by 20 endoscopists, certified by the Japan Gastroenterological Endoscopy Society, to ensure that detected neoplastic lesions were included. The endoscopist annotated the boundary of the neoplastic lesion in at least one frame, after which the CRO annotated the same lesion boundary across the remaining frames of the video clip (Figure 3). The neoplastic lesion datasets consisted of video clips with annotated neoplastic lesions. The non‐neoplastic lesion datasets comprised video clips without annotated neoplastic lesions and those with neoplastic lesions, excluding the area where the neoplastic lesions were annotated. The finalized neoplastic lesion datasets were used as the gold standard for neoplastic lesions. Similarly, finalized non‐neoplastic lesion datasets were used as the gold standard to confirm the absence of such lesions, with endoscopists verifying these datasets to ensure their accuracy.

### 2.4 Evaluation of the Performance of the CAD EYE

The performance of the CAD EYE in endoscopic detection was evaluated using a dataset by comparing the rectangular area output by the CAD EYE with the gold standard annotation of neoplastic lesions. As the CAD EYE is designed to display a rectangle as the detection flag, successful lesion detection was defined as the presence of a rectangular frame overlapping, even partially, with the annotation in at least three consecutive frames within the dataset. Detection failure was defined as fewer than three consecutive frames in which a rectangular flag overlapped with an annotated lesion or the absence of any detection. Data managers, who were not involved in the annotation process, independently reviewed the detection results to classify detections as successful or failed based on the presence of the rectangular flag. The primary endpoint was the sensitivity of the CAD‐EYE for detecting ESCC and GN. Sensitivity was defined as the number of datasets in which lesions were successfully detected, divided by the total number of neoplastic lesion datasets. Specificity was defined as the number of datasets without a detection flag, divided by the total number of non‐neoplastic lesion datasets. In addition, flags in a dataset in a post hoc analysis. It was defined as the number of datasets without a detection flag, divided by the number of datasets without a detection flag and the number of detection flags in the non‐neoplastic lesion datasets.

### 2.5 Sample Size Calculation and Statistics

Based on the reported detection sensitivities for ESCC and GN, the sensitivity of the CAD EYE was assumed to be 80% [22, 23, 24]. The required sample size was calculated as 246 using the Wald test for a 95% confidence interval (CI) with an error of ±5%. All statistical analyses were performed using SAS software (version 9.4; SAS Institute Inc., Cary, NC, USA). The sample size was determined before the CRO created an image dataset.

## 3 Results

**FIGURE 4:**
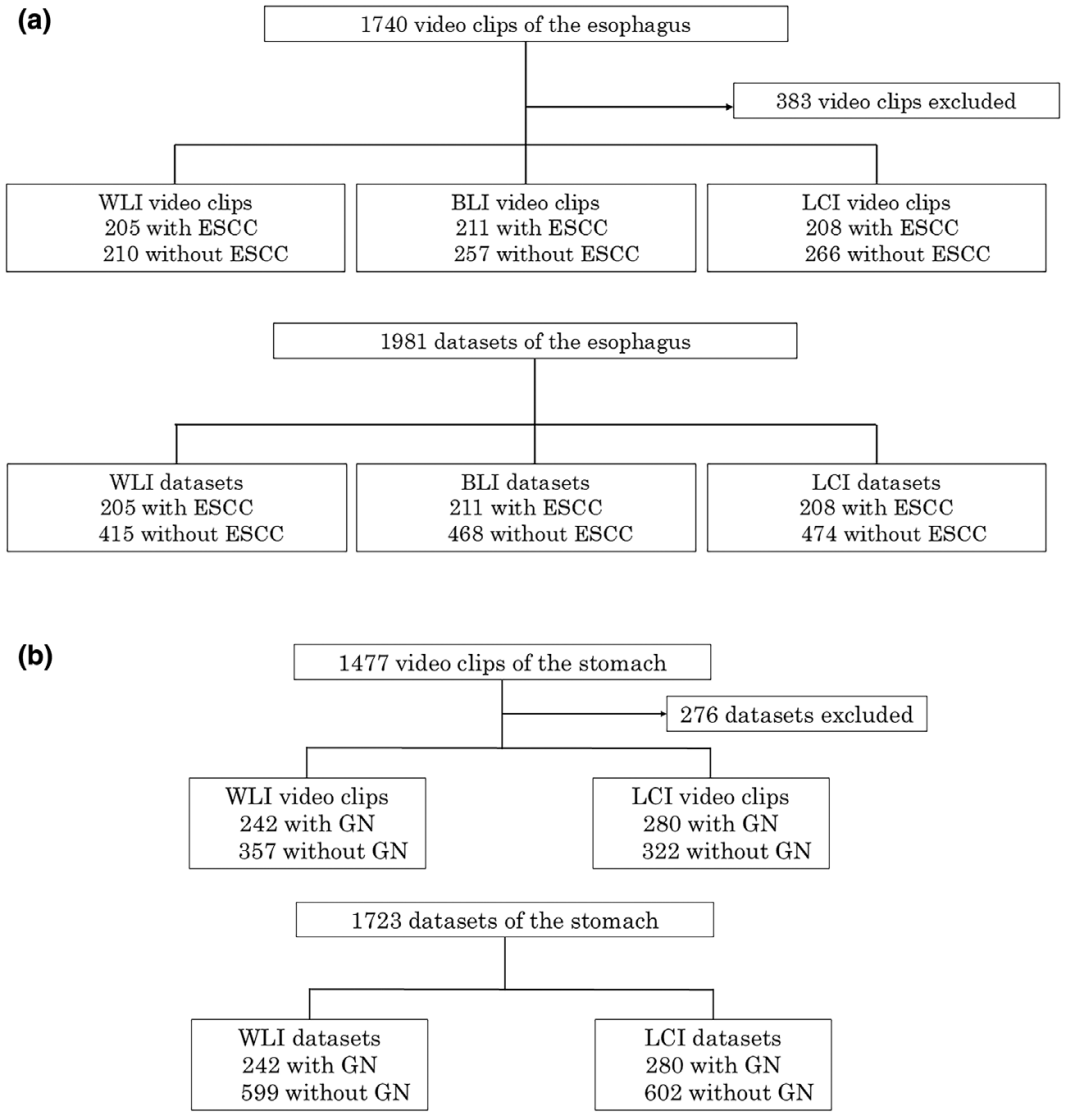
Study flow. (a) Study flow for the esophagus. The datasets without esophageal squamous cell carcinoma (ESCC) comprised of video clips without annotated ESCCs and ones with ESCCs excluding the area where the ESCCs were annotated. (b) Study flow for the stomach. The datasets without gastric neoplastic lesion (GN) comprised of video clips without annotated GNs and ones with GNs excluding the area where the GNs were annotated.

A total of 3217 video clips (1740 from the esophagus and 1477 from the stomach) were obtained from 14 institutions between May 2019 and May 2021. Of these, 383 esophageal video clips and 276 gastric video clips were excluded from the analysis because no lesion was identified by the endoscopists in the video clips (239 esophagus and 176 stomach) or the target lesion was not histologically confirmed as ESCC or GN (144 esophagus and 100 stomach) (Figure 4).

For the esophageal analysis, we obtained 205 and 210 WLI, 211 and 257 BLI and 208 and 266 LCI video clips with and without ESCC, respectively. The median lesion sizes in the WLI, BLI, and LCI groups were 15.5, 17.0, and 17.0 mm, respectively. Most lesions were histologically confirmed as pT1a ESCC confined to the mucosa (Table 1). We used 205 datasets with ESCC and 415 datasets without ESCC in WLI, 211 with ESCC and 468 without ESCC in BLI, and 208 with ESCC and 474 without ESCC in LCI, respectively (Figure 4a). The sensitivity and specificity were 85.9% (176/205) and 93.3% (387/415), respectively, in the WLI group; 97.6% (206/211) and 92.9% (435/468) in the BLI group; and 96.6% (201/208) and 93.2% (442/474) in the LCI group (Table 2). The specificity in the detection flag was not evaluated because of no multiple flags in a no‐neoplastic lesion dataset. Table 3 shows the performance of ESCC detection according to lesion characteristics. The sensitivities for ESCC lesions ≤ 10 mm were 78.7%, 77.3%, and 92.0% in the WLI, BLI, and LCI groups, respectively. The sensitivities for pT1a ESCC were 85.3%, 97.3%, and 97.2% in the WLI, BLI, and LCI groups, respectively.

**TABLE 1:** Lesion characteristics of esophageal squamous cell carcinoma.

	WLI		BLI		LCI	
Number of lesions	205		211		208	
Tumor location, *n* (%)						
Ce‐Ut	34	16.6%	33	15.6%	32	15.4%
Mt	133	64.9%	129	61.1%	131	63.0%
Lt‐Jz	38	18.5%	48	22.8%	45	21.6%
Unknown	0	0%	1	0.5%	0	0%
Macroscopic type, *n* (%)						
0–IIa	32	15.7%	20	9.5%	31	14.9%
0–IIb	60	29.4%	69	32.7%	60	28.8%
0–IIc	106	52.0%	119	56.4%	111	53.4%
Unknown	7	3.4%	3	1.4%	6	2.9%
**Lesions size, mm**						
Median (range), mm	15.5	(3–90)	17.0	(3–80)	17.0	(3–90)
1 ~ 10	61	29.9%	22	10.4%	25	12.0%
11 ~ 20	64	31.4%	88	41.7%	81	38.9%
20 ~	68	33.3%	90	42.7%	93	44.7%
Unknown	12	5.9%	11	5.2%	9	4.3%
Depth of invasion, *n* (%)						
pT1a	170	82.9%	186	88.1%	177	85.1%
pT1b	24	11.7%	20	9.5%	18	8.7%
> pT2	5	2.4%	1	0.5%	5	2.4%
Unknown	6	2.9%	4	1.9%	8	3.8%

Abbreviations: BLI, blue laser/light imaging; LCI, linked color imaging; WLI, white‐light imaging.

**TABLE 2:** Performance of esophageal squamous cell carcinoma detection.

Mode	Sensitivity [95% CI]	Specificity [95% CI]
WLI	85.9% (176/205) [81.1%–90.6%]	93.3% (387/415) [90.8%–95.7%]
BLI	97.6% (206/211) [95.6%–99.7%]	92.9% (435/468) [90.6%–95.3%]
LCI	96.6% (201/208) [94.2%–99.1%]	93.2% (442/474) [91.0%–95.5%]

Abbreviations: BLI, blue laser/light imaging; CI, confidence interval; LCI, linked color imaging; WLI, white‐light imaging.

**TABLE 3:** Esophageal squamous cell carcinoma detection according to the lesion characteristics.

Number of cases	WLI				BLI				LCI			
	Detected		Not detected		Detected		Not detected		Detected		Not detected	
	176		29		206		5		201		7	
Morphology, *n* (%)												
0–I	0	N.A.	0	N.A.	0	N.A.	0	N.A.	0	N.A.	0	N.A.
0–IIa	27	84.4%	5	15.6%	18	90.0%	2	10.0%	28	90.3%	3	9.7%
0–IIb	50	83.3%	10	16.7%	68	98.6%	1	1.4%	57	95.0%	3	5.0%
0–IIc	95	89.6%	11	10.4%	118	99.2%	1	0.8%	111	100.0%	0	0.0%
Unknown	4		3		2		1		5		1	
Size, *n* (%)												
1 ~ 10 mm	48	78.7%	13	21.3%	17	77.3%	5	22.7%	23	92.0%	2	8.0%
11 ~ 20 mm	57	89.1%	7	10.9%	88	100.0%	0	0.0%	80	98.8%	1	1.2%
20 mm ~	59	86.8%	9	13.2%	90	100.0%	0	0.0%	89	95.7%	4	4.3%
Unknown	12				11		0		9		0	
Location, *n* (%)												
Ce‐Ut	27	79.4%	7	20.6%	29	87.9%	4	12.1%	30	93.8%	2	6.3%
Mt	119	89.5%	14	10.5%	129	100.0%	0	0.0%	127	96.9%	4	3.1%
Lt‐Jz	30	78.9%	8	21.1%	47	97.9%	1	2.1%	44	97.8%	1	2.2%
Unknown	0		0		1		0		0		0	
Depth of invasion, *n* (%)												
pT1a	145	85.3%	25	14.7%	181	97.3%	5	2.7%	172	97.2%	5	2.8%
pT1b	22	91.7%	2	8.3%	20	100.0%	0	0.0%	18	100.0%	0	0.0%
> pT2	4	80.0%	1	20.0%	1	100.0%	0	0.0%	4	80.0%	1	20.0%
Unknown	5		1		4				7		1	

Abbreviations: BLI, blue laser/light imaging; CI, confidence interval; LCI, linked color imaging; N.A., not available; WLI, white‐light imaging.

For the GN analysis, we analyzed 242 WLI and 280 LCI video clips with neoplastic lesions, along with 357 WLI and 322 LCI video clips without neoplastic lesions. The median lesion size in the WLI and LCI groups was 10.5 and 10.0 mm, respectively. Most lesions were histologically diagnosed as mucosal EGC (Table 4). We used 242 datasets with GN and 599 datasets without GN in WLI, 280 with GN and 602 without GN in LCI, respectively (Figure 4b). The sensitivity and specificity of GN detection were 95.5% (231/242) and 86.1% (516/599), respectively, in the WLI group, and 93.9% (263/280) and 94.4% (568/602) in the LCI group (Table 5). There were five and three multiple detection flags in five WLI and two LCI non‐GN datasets. Thus, the specificity in the detection flags was 85.4% (516/604) and 93.9% (568/605) in the WLI and LCI groups, respectively (Table 5). Table 6 shows the performance of GN detection according to the lesion characteristics. The sensitivities for GN lesions ≤ 10 mm were 95.8% in the WLI group and 93.2% in the LCI group. The sensitivities of pT1a EGC were 93.8% in the WLI group and 92.4% in the LCI group.

**TABLE 4:** Lesion characteristics of gastric neoplasm.

	WLI		LCI	
Number of cases	242		280	
Macroscopic type, *n* (%)				
0–I	5	2.1%	7	2.5%
0–IIa	90	37.2%	110	39.3%
0–IIb	9	3.7%	14	5.0%
0–IIc	128	52.9%	137	48.9%
0–III	1	0.4%	1	0.4%
Unknown	9	3.7%	11	3.9%
Location (axial direction), *n* (%)				
U	29	12.0%	24	8.6%
M	118	48.8%	123	43.9%
L	95	39.2%	128	45.7%
Unknown	0		5	1.8%
Location (circumferential), *n* (%)				
Anterior wall	47	19.4%	48	17.1%
Posterior wall	56	23.1%	58	20.7%
Lesser curvature	66	27.3%	92	32.9%
Greater curvature	60	24.8%	65	23.2%
Unknown	13	5.4%	17	6.1%
Lesion size, *n* (%)				
Median (range), mm	10.5	(2–70)	10.0	(2–70)
1 ~ 10 mm	119	49.2%	147	52.5%
11 ~ 20 mm	76	31.4%	89	31.8%
21 mm ~	43	17.8%	38	13.6%
Unknown	4	1.7%	6	2.1%
Histology, *n* (%)				
Adenoma	26	10.7%	31	11.1%
Adenocarcinoma	216	89.3%	249	88.9%
Depth of invasion, *n* (%)				
pT1a	162	75.0%	197	81.1%
pT1b	39	18.1%	35	14.0%
> pT2	8	3.7%	4	2.1%
Unknown	7	3.2%	7	2.9%

Abbreviations: LCI, linked color imaging; WLI, white‐light imaging.

**TABLE 5:** Performance of gastric neoplasm detection.

Mode	Sensitivity [95% CI]	Specificity [95% CI]	Specificity in the detection flag [95% CI]
WLI	95.5% (231/242) [92.8%–98.1%]	86.1% (516/599) [83.4%–88.9%]	85.4% (516/604) [82.6%–88.2%]
LCI	93.9% (263/280) [91.1%–96.7%]	94.4% (568/602) [92.5%–96.2%]	93.9% (568/605) [92.0%–95.8%]

Abbreviations: CI, confidence interval; LCI, linked color imaging; WLI, white‐light imaging.

**TABLE 6:** Gastric neoplasm detection according to lesion characteristics.

Number of cases	WLI				LCI			
	Detected		Not detected		Detected		Not detected	
	231		11		263		17	
Location (axial direction), *n* (%)								
U	27	93.1%	2	6.9%	20	83.3%	4	16.7%
M	112	94.9%	6	5.1%	118	95.9%	5	4.1%
L	92	96.8%	3	3.2%	120	93.8%	8	6.3%
Uknown	0		0		5		0	
Location (circumferential), *n* (%)								
Anterior wall	44	93.6%	3	6.4%	45	93.8%	3	6.3%
Posterior wall	55	98.2%	1	1.8%	51	87.9%	7	12.1%
Lesser curvature	63	95.5%	3	4.5%	89	96.7%	3	3.3%
Greater curvature	56	93.3%	4	6.7%	61	93.8%	4	6.2%
Uknown	13		0		17		0	
Macroscopic type, *n* (%)								
0–I	5	100.0%	0	0.0%	7	100.0%	0	0.0%
0–IIa	83	92.8%	7	7.8%	101	91.8%	9	8.2%
0–IIb	8	88.9%	1	11.1%	13	92.9%	1	7.1%
0–IIc	125	97.7%	3	2.3%	130	94.9%	7	5.1%
0–III	1	100.0%	0	0.0%	1	100.0%	0	0.0%
Unknown	9		0		11		0	
Size, *n* (%)								
1 ~ 10 mm	114	95.8%	5	4.2%	137	93.2%	10	6.8%
11 ~ 20 mm	72	94.7%	4	5.3%	85	95.5%	4	4.5%
21 mm ~	41	95.3%	2	4.7%	35	92.1%	3	7.9%
Unknown	4		0		6		0	
Histology, *n* (%)								
Adenoma	26	96.2%	1	3.8%	31	100.0%	0	0.0%
Adenocarcinoma	216	95.4%	10	4.6%	232	93.6%	17	6.4%
Depth of invasion, *n* (%)								
pT1a	152	93.8%	10	6.2%	182	92.4%	15	7.6%
pT1b	39	100.0%	0	0.0%	33	94.3%	2	5.7%
> pT2	8	100.0%	0	0.0%	4	100.0%	0	0.0%
Unknown	7		0	6	7	0	0	

Abbreviations: LCI, linked color imaging; WLI, white‐light imaging.

## 4 Discussion

This multicenter study investigated the performance of the CAD EYE system for detection of ESCC and GN. This study demonstrated the high sensitivity of the CAD EYE for detecting upper gastrointestinal neoplasms, with the lower limit of the 95% confidence interval for sensitivity exceeding 80%, based on the datasets from 14 institutions across Japan. Sensitivity met the primary endpoint; despite a smaller‐than‐anticipated sample size, as more datasets were excluded from the analysis than expected.

Several similar image‐ and video‐based studies have investigated AI‐assisted endoscopic detection of upper gastrointestinal neoplastic lesions [22, 23, 24]. Some randomized controlled trials have shown that AI‐assisted endoscopy is superior to standard endoscopy in detecting EGC and ESCC, showing promising efficacy for real‐time AI‐assisted endoscopy [25, 26, 27, 28]. However, AI‐assisted esophagogastroscopy using BLI and LCI remains underdeveloped. Few reports have investigated the potential of CAD combined with IEE and compared its performance to WLI for detecting upper gastrointestinal neoplasms.

In terms of sensitivity for ESCC and specificity for GN, IEE may improve the performance of CAD EYE compared with standard WLI. Particularly, the performance of the CAD EYE may be insufficient for detecting ESCC using WLI in clinical practice. The current expert statement recommends the use of IEE for detecting ESCC, based on the results of randomized controlled trials [29, 30]. Japanese guidelines state that the use of IEE for detecting EGC remains under discussion due to weak evidence [4]. Nevertheless, a recent multicenter randomized controlled trial (LCI‐FIND) demonstrated that LCI was more effective than WLI in detecting neoplastic lesions in the pharynx, esophagus, and stomach [18]. Consequently, using IEE could facilitate the development of AI‐assisted endoscopy, improving its performance in detecting upper gastrointestinal neoplasms, as demonstrated by endoscopists in clinical trials.

Regarding the use of CAD‐EYE in clinical practice, the sensitivities for pT1a lesions were sufficiently high for ESCC and GN, except for WLI in ESCC, as mentioned above. Also, the sensitivity for small GN lesions (≤ 10 mm) was over 90%. These findings suggest that CAD‐EYE could be effectively utilized in clinical practice. However, further improvement is desirable for detecting small ESCC in BLI.

This study has several limitations. The main limitation is its retrospective design, which could introduce selection bias. Endoscopists performed esophagogastroscopy, with the choice of WLI, BLI, or LCI determined at their discretion. Sensitivity and specificity were analyzed using the dataset extracted from esophagogastroscopy videos by the CRO. This approach may introduce selection bias, resulting in various patients' backgrounds, the inclusion of only evaluable endoscopic images, and excluding those of poor quality, such as images with poor insufflation, light reflection, defocus, or mucus. Consequently, the results of this study may not fully reflect actual clinical practice. Further investigation is required to assess real‐time lesion detection and evaluate the diagnostic yield for inexperienced endoscopists, with or without the use of CAD‐EYE. The ongoing development of AI technology is anticipated to enhance lesion detection quality by updating the training dataset.

In conclusion, the CAD EYE prototype demonstrated high sensitivity for detecting ESCC and GN, highlighting its potential as a promising AI tool for clinical applications.

## Author Contributions

**Seiichiro Abe:** drafting of the article, acquisition, analysis, and final approval of the article. **Yoshiyasu Kitagawa:** acquisition and interpretation of data, and revising the draft critically for important intellectual content. **Waku Hatta:** acquisition and interpretation of data, and revising the draft critically for important intellectual content. **Takao Maekita:** acquisition and interpretation of data, and revising the draft critically for important intellectual content. **Motohiko Kato:** acquisition and interpretation of data, and revising the draft critically for important intellectual content. **Akihito Nagahara:** acquisition and interpretation of data, and revising the draft critically for important intellectual content. **Hiroyuki Osawa:** acquisition and interpretation of data, and revising the draft critically for important intellectual content. **Osamu Dohi:** acquisition and interpretation of data, and revising the draft critically for important intellectual content. **Hirotaka Nakashima:** acquisition and interpretation of data, and revising the draft critically for important intellectual content. **Kazuhiro Furukawa:** acquisition and interpretation of data, and revising the draft critically for important intellectual content. **Shiro Oka:** acquisition and interpretation of data, and revising the draft critically for important intellectual content. **Tomoko Yokoyama:** acquisition and interpretation of data, and revising the draft critically for important intellectual content. **Toru Ito:** acquisition and interpretation of data, and revising the draft critically for important intellectual content. **Ichiro Oda:** conception, acquisition, and interpretation of data; revising the draft critically for important intellectual content; and final approval of the article.

## Ethics Statement

This study was approved by the institutional review boards of all participating facilities; written informed consent for this study was obtained from all patients.

## Conflicts of Interest

S.A. has received research grants, honoraria, and travel from Fujifilm Corporation. Y.K. has received research grants, honoraria, and travel from Fujifilm Corporation. W.H. has received research grants, honoraria, and travel from Fujifilm Corporation. T.M. has received research grants from Fujifilm Corporation. M.K. has received research grants, contract, honoraria, and travel from Fujifilm Corporation. A.N. has received research grants and honoraria from Fujifilm Corporation. H.O. has received research grants from Fujifilm Corporation, honoraria from Fujifilm Corporation, Fujifilm Medical Corporation, and Takeda Pharmaceuticals. O.D. has received research grants from Fujifilm Corporation. H.N. has received research grants and honoraria from Fujifilm Corporation. K.F. has received research grants and honoraria from Fujifilm Corporation. S.O. has received research grants and honoraria from Fujifilm Corporation. T.Y. has stock and has received other services from Fujifilm Corporation. T.I. has received research grants and honoraria from Fujifilm Corporation. I.O. has received research grants and honoraria from Fujifilm Corporation.
